# The effect of exercise on academic fatigue and sleep quality among university students

**DOI:** 10.3389/fpsyg.2022.1025280

**Published:** 2022-10-21

**Authors:** Wenjing Li, Jianing Chen, Mingping Li, Andrew P. Smith, Jialin Fan

**Affiliations:** ^1^School of Psychology, Shenzhen University, Shenzhen, China; ^2^Center for Mental Health, Shenzhen University, Shenzhen, China; ^3^School of Psychology, Cardiff University, Cardiff, United Kingdom

**Keywords:** stress, academic fatigue, sleep quality, college students’ health, exercise

## Abstract

**Background:**

Routine academic events may cause fatigue and impair sleep quality. This research aimed to examine the prevalence and risk factors for academic fatigue among college students and its adverse effects on well-being and sleep. A brief exercise intervention was also evaluated.

**Methods:**

A total of 864 college students (33.5% female) filled out self-reported questionnaires with few open-ended questions. Fatigue and sleep quality were assessed using the translated version of the Smith Well-being Questionnaire and the Chinese version of the Pittsburgh Sleep Quality Index (PSQI). Spearman correlations, logistic regression analysis, and *t*-tests were used to test the hypotheses. In a second study, 29 female participants took part in an exercise intervention aimed at reducing fatigue and improving sleep.

**Results:**

Among the effective respondents, nearly 40% reported higher academic-related fatigue, and a few reported high-quality sleep. Negative coping styles, workload, stress, and disturbed surroundings had a significant positive predictive effect on academic fatigue. In addition, adverse consequences of fatigue were found for physical health and academic-life balance, and a significant, positive relationship was observed between the degree of fatigue and PSQI score (*p* < 0.01). The exercise study showed some beneficial effects of the intervention for both sleep and fatigue outcomes.

**Conclusion:**

Fatigue is common and widely reported among Chinese college students, and it may have a major negative impact on their health. Increased awareness of daily academic fatigue and its impact on college students is important for individuals, schools, and society. Exercise may be a simple way to improve sleep and reduce fatigue.

## Introduction

Fatigue is a psychophysiological state of decreased ability and effectiveness (Simonson and Weiser, 1976; Phillips, 2015) that typically occurs during highly demanding tasks requiring long periods of mental and physical input (van der Linden et al., 2003; Dolezal et al., 2017), and it may have a great impact on the individual’s performance and well-being, including sleep-disturbance (Rodrigue et al., 2010; Hockey, 2013; Weaver and Barger, 2019). The existing research on fatigue and sleep issues has primarily focused on the workplaces that are known to cause fatigue (e.g., road transport, healthcare, aviation, etc. see Dawson et al., 2021 for a review). Academic responsibilities during college provide numerous challenges and lead to fatigue becoming an everyday occurrence for many students. A typical workplace may cause fatigue (Salmela-Aro et al., 2008). However, the academic environment may be an often-overlooked part of the picture. In the present study, we were interested in a more detailed profile of academic-related fatigue among college students and its causes and effects.

With the increasingly intense competition for employment, many college students are under greater academic pressure to achieve outstanding performance at school. Particularly in East Asia, there is a strong culture in which academic achievements stand for social acceptance and status (Huang and Chou, 2010; Park et al., 2021). To make themselves more employable, students invest a lot of time in their studies. It’s no wonder then that they suffer from academic fatigue due to enduring hours of lessons, examinations, competition, and schoolwork with their heads buried in textbooks (Archer and Lamnin, 1985; McDonald, 2001; Hughes, 2004, 2005; Likhon, 2021). The demands-resources-individual effects (DRIVE) model is one model in the field of occupational fatigue research used to analyze the key elements of the stress process (Mark, 2008). It has provided valuable insight into the factors that contribute to increased fatigue, including (1) work demands (e.g., workload, extrinsic effort), (2) job characteristics (e.g., social support, rewards), and (3) individual differences (e.g., personality trait, lifestyle; Ranjan and Prasad, 2017). College students need to attend regular and structured classes and complete their assignments every day, which is comparable with the work of formal employees. That is, students’ main activities at school can be considered a form of work, so daily academic fatigue occurs due to coping with academic stress accrued from routine experiences (Schaufeli et al., 2002; Salmela-Aro et al., 2009). However, there is scanty research reporting academic-related fatigue and its associated factors. For this reason, the current study aims to estimate the prevalence of fatigue and identify risk factors among university students in China using the DRIVE model as a framework.

Health problems of young people have always been a concern. Fatigue is often accompanied by a lack of physical and mental strength and a decline in motivation (Boksem et al., 2006), which could hinder the healthy growth of young people. The DRIVE model attempted to account for the associations between subjective fatigue perceptions and health outcomes and demonstrated that central adverse effects of fatigue are related to poor health conditions, illness, lack of work-life balance, and impaired performance (Mark and Smith, 2008; Smith, 2021).

However, an overlooked negative outcome of stress is decreased sleep quality. Daily academic fatigue inevitably has repercussions on college students’ sleep. It has been shown that people are particularly susceptible to inadequate sleep and report significant impairments in sleep quantity and quality under higher daily stress (Pilcher et al., 1997; Lund et al., 2010; Becker et al., 2018). With an increase in daytime pressure, individuals are more likely to feel tired, and this, in turn, causes difficulties in falling asleep, maintaining sleep, and ensuring sleep quality (Yang et al., 2014; Wang and Bíro, 2021). Several studies on college students have linked academic stress to shorter sleep times during exam periods (e.g., Harsh et al., 2007; Zhang et al., 2017; Campbell et al., 2018). Poor sleep can lead to subsequent fatigue. These studies all corroborate that college students are at risk for poor sleep during exam periods. Nevertheless, few studies target fatigue in the daily life of college students and test its impact directly on sleep quality. Accordingly, the secondary aim of this study was to investigate the relationship between daily academic fatigue and sleep quality in college students.

Fatigue impairs young people’s attention, leading to slowed thinking and poor perception (Boksem et al., 2005), and lack of sleep impairs adolescents’ academic ability, social–emotional and behavioral functioning, and academic performance (Castilhos Beauvalet et al., 2017; Elfering et al., 2020), adversely affecting college students’ learning efficiency and academic outcomes. These negative consequences have prompted researchers to explore effective interventions to reduce the effects of fatigue and improve sleep quality in young people.

Exercise as a non-pharmacological means has low cost, low side effects, and convenience. Numerous studies have shown that exercise interventions can effectively reduce students’ fatigue and improve individual sleep quality (Erlacher et al., 2015; Brand et al., 2014; Kredlow et al., 2015; Lang et al., 2016; Ezati et al., 2020). Psychological and physiological mechanisms may explain the effect of exercise on academic fatigue. Students may reduce fatigue by exercising in a way that creates “psychological separation” (Otto and Smits, 2011; Sonnentag, 2012). Exercise allows students to temporarily divert their attention while enhancing their physical fitness and engaging in learning in a more relaxed way. People who exercise regularly also have less stress, anxiety, and depression (Driver and Taylor, 2000). In addition, it has long been shown that people who exercise regularly have fewer self-reported sleep problems and are less likely to be sleepy during the day than sedentary people (Dolezal et al., 2017). Exercise has a moderate to a large positive effect on all subscales of the Pittsburgh sleep quality index (PSQI). Also, long-term exercise increases total sleep time and sleep efficiency to some extent and has a small to moderate effect on sleep latency (SOL) (Kredlow et al., 2015).

Research on academic fatigue and sleep in young adults or college students is still scarce, with most studies focusing on sick populations, and there is still some uncertainty about the effect of exercise on sleep. Some researchers have been skeptical about the role of exercise in affecting sleep, the study found that exercise appeared to have little effect on improving sleep, and suggesting that the positive effects of exercise have been exaggerated (Coleman et al., 2012). In a review of exercise and sleep, it was stated that four researchers did not find any difference in sleep from exercise, and one study even claimed that exercise had a negative effect on sleep (Dolezal et al., 2017). Furthermore, sedentary behavior and physical activity were not necessarily associated with sleep quality in all populations. The researchers noted that the relationship between physical activity and self-reported sleep duration was dependent on age and gender (McClain et al., 2014). Differences in the exercise regimens studied (e.g., aerobic or anaerobic, intensity, duration) and interactions between individual characteristics (e.g., health, age, and gender) make the experimental evidence for a sleep-enhancing effect of exercise less supportive. However, at the same time, some researchers have commented that, regardless of the pattern and intensity of activity, exercise improves sleep efficiency and duration, especially in people with medical conditions (Dolezal et al., 2017).

Gender differences also need to be taken into account when studying academic stress and sleep problems. A study showed that women, but not men, experienced a significant reduction in fatigue after a 6-month exercise intervention (Surakka et al., 2004). The results from a survey showed that the average score of females’ perception of academic stress is higher than that of men (Calaguas, 2011). Meanwhile, studies have found that women have a higher prevalence of poor sleep quality than men (Dolezal et al., 2017; Bani-issa et al., 2018), and another study has shown that female college students went to bed and woke up early, had a long sleep latency, woke up more often, and had poor sleep quality than males (Tsai and Li, 2004). All these show that female college students are more typical poor sleepers. It is important to conduct research on academic stress and sleep for the female college student population. Therefore, the third aim of this study was to find out whether different forms and intensities of exercise can have a positive effect on academic fatigue and sleep in female college students. In addition, most studies on exercise interventions for fatigue and sleep have been longer than 8 weeks, and we wanted to investigate whether a short-term intervention (e.g., 1 week) could also change academic-related fatigue and sleep in college students.

Routine academic events may cause fatigue in college students. However, there are very few studies that have systematically evaluated the prevalence and predictors of such fatigue. Also, the associations of sleep problems with the various aspects of fatigue in college students remain unclear. There is also the question of whether exercise is an effective tool to help female university students improve their daily academic fatigue and sleep quality. The present study sought to investigate (a) whether college students exhibit academic-related fatigue, (b) the contribution of academic events to the explanation of academic fatigue by individual differences, school resources, social support, as well as workload, and (c) the interaction between fatigue, health, and sleep problems in young adults in China, and (d) The extent to which exercise reduces academic fatigue and improves sleep among female university students.

Two studies were carried out to address these questions. Study 1 was a survey of a sample of college students. Previous studies have revealed that academic pressure is one of the most influential factors in college students’ daily lives (El Ansari et al., 2014) but have not reported clear causes and results of this impact. Thus, we used the DRIVE model as the research framework and conducted our assessment using a multi-dimension questionnaire, which is suitable for the investigation of the causes and results of workload and fatigue. Specifically, it was hypothesized that (1) the fatigue experienced among college students would be significantly associated with academic events, individual differences (i.e., personality, self-efficacy, coping style, and lifestyle), school resources (i.e., studying environment), and social support, and academic workload. Regarding the negative outcomes of academic fatigue, it was hypothesized that (2) academic-related fatigue is not only associated with negative mental well-being and physical illness but also with sleep problems. In terms of gender differences, consistent with previous studies, it was hypothesized that (3) female university students would have higher levels of academic fatigue and lower quality sleep compared to males.

In study 2, female university students with high levels of fatigue and poor sleep quality were selected from responses to the survey given in study 1 and given a 6-day short-term exercise intervention in which they were asked to perform a total of 30 min of moderate intensity, any type of physical activity each day. In addition, sleep-related indicators were measured by actigraphy over the six-day period, and changes in the participants’ perceptions of academic fatigue before and after the exercise intervention were measured by an online questionnaire. It was hypothesized that: Short-term exercise would be effective in decreasing fatigue and improving sleep efficiency and quality of sleep in a typical group of female university students with academic fatigue and sleep problems.

## Study 1

To investigate the risk factors for academic stress and its correlation with fatigue among college students, as well as the correlation between the consequence of academic fatigue and sleep, we used the SWELL Questionnaire (Smith Wellbeing Questionnaire), the Pittsburgh Sleep Quality Index (Buysse et al., 1989) and academic-related questions were combined and developed into an online questionnaire that was widely distributed to college students. At the same time, this study wanted to compare the findings of previous studies that used male college students, female college students have significantly higher levels of academic anxiety (Calaguas, 2011) and lower sleep quality (Bani-issa et al., 2018) also hold in this study. The questionnaires collected were also used to screen a group of female college students who met the requirements for the subsequent exercise intervention experiment.

### Methods

#### Participants

We used G*Power to calculate the sample size, effect size *f* = 0.3, α = 0.05, 1-β = 0.8, and calculated the required sample size to be 82. Based on the calculated subject size, 846 students from Shenzhen University were recruited as participants in order to improve the scientific validity of the study findings. The participants were aged between 18 and 28 years recruited using posters on campus and an online forum. All were enrolled in the survey on a voluntary basis, with the option of withdrawing at any point without any disadvantages. After excluding respondents who answered too quickly or too slowly, response rest, and random or regular answers, a total of 756 college students (32.5% female, *M*_age_ = 20.55 years, *SD* = 1.35 years) were included in the full analysis.

#### Task and procedure

The design of this study was reviewed and approved by the Research Ethics Committee of the School of Psychology, Shenzhen University. Before data collection, each participant read and approved the online informed consent statement. During the testing sessions, participants filled out a series of online self-reported questionnaires consisting of (1) respondents’ demographic characteristics (e.g., gender, age, and grades) and recent academic assignments, (2) self-statements about the cause and outcomes of daily academic fatigue, and (3) self-reported measures of sleep quality. The survey took between 15 and 20 min to complete. There was no monetary compensation for participation in the study, but those who accomplished the task entered a lucky draw, which gave them a chance to win campus postcards, sleeping masks, or other gifts.

#### Measurements

##### Smith well-being questionnaire

Students’ academic fatigue was assessed using the Smith Well-being Questionnaire (SWELL; Smith and Smith, 2017), which was established based on the DRIVE model for a comprehensive range of outcomes related to health and performance efficiency. This is a single-item scale, the items of SWELL are based on the single-item design of the Well-being Process Questionnaire (WPQ), and previous researchers have compared single-item and multiple items (WPQ) and have confirmed the validity and reliability of this single-item measure of well-being (Williams and Smith, 2016). In addition, previous studies have modified the questionnaire for use with students and found that it is suitable for the assessment of students’ fatigue (Alharbi and Smith, 2019; Nor and Smith, 2019). The participants were asked to respond to 37 self-report items, within the last 3 months, most of which were on a 10-point scale, with the rest as yes/no questions.

The self-statements consisted of two blocks: the predictors and the outcomes of academic fatigue. Questions for the predictors block measured the factors that led to fatigue, including academic characteristics (e.g., “Overall, how stressful is your academic life?”), individual differences (e.g., “To what extent do you try to positively cope with stress, such as trying to solve problems or seeking social support?”), social support (e.g., “I feel that I have the social support I need.”), and the learning environment (e.g., “Are you exposed to noise in your learning environment?”). Questions for the results block measured well-being outcomes included physical health (e.g., “Do you suffer from musculoskeletal disorders such as back pain or headaches?”), positive well-being (e.g., “To what extent do you have positive feelings at work, such as feel happiness, excitement, relaxation, and sociability?”), academic-life balance (e.g., “Do you find that your work interferes with other aspects of your life?”), and academic performance, including academic efficacy and academic satisfaction (e.g., “How efficiently do you learn?”). The variables mentioned in the results of the follow-up study were measured using the SWELL questionnaire.

Both forward and reverse translations were used to translate this survey into Chinese. The forward translation of SWELL from English to Chinese was carried out by two researchers who were fluent in both languages. The backward translation was then carried out by an impartial translator who was unaware of the assessment. Before making any last-minute changes to the questionnaire, the original English and the reverse translated English versions were compared and any differences were reviewed.

##### Pittsburgh sleep quality index

Sleep during the past month was assessed using the PSQI, one of the most commonly used tools for sleep quality assessment (Buysse et al., 1989). The PSQI is a self-rated questionnaire that enables to assess of overall sleep quality with 19 items generating seven components: (1) subjective sleep quality, (2) sleep latency, (3) sleep duration, (4) habitual sleep efficiency, (5) sleep disturbances, (6) use of sleeping medication, and (7) daytime dysfunction. Sleep duration was recorded as hours: minutes, and sleep latency was measured in minutes. Each component was coded and scored from 0 to 3 and then summed into a global PSQI score, with higher scores indicating poorer sleep quality. Cronbach’s α was 0.83 when it was developed (Buysse et al., 1989) and 0.88 in this study.

#### Statistical analysis

Data analysis was performed using IBM SPSS for Mac, version 25. Descriptive statistics were first calculated to examine the frequency of responses, and a series of bivariate correlations among the variables were examined using Pearson correlation tests. Next, hierarchical multiple analysis was performed to uncover which factors predicted academic fatigue. The variables were dichotomized into two broad and distinct groups of groups (usually by using the thresholds, e.g., above thresholds for a high group), and an independent *t*-test was conducted to assess whether well-being outcomes and academic performance were associated with academic fatigue. Finally, the association between academic fatigue and sleep quality was assessed. Correlation analysis and independent *t*-tests were conducted to assess the sleep quality (according to the self-report questions on PSQI) according to those with high and low levels of academic fatigue (based on the single item score of SWELL questionnaires).

### Results

#### Descriptive statistics

Among the effective respondents, there were 510 men and 246 women, and most participants were in their first year (65.7%). Two-thirds of participants scored themselves as having negative personalities (63.4%), and about half thought they had unhealthy lifestyles (53.3%). The mean fatigue score was in the mid-range (4.98 ± 2.45), and 41% of students reported high fatigue (above the median). Higher fatigue levels were reported by participants in response to challenges, such as applications for studying abroad (66.7%), taking part in the postgraduate entrance exams (64.6%), and preparing for graduation theses (55.9%) when applying median split to fatigue. The mean sleep duration of the participants was 6. 85 ± 0.95 h, and the average PSQI global score was 6.93 ± 3.17, with the minority of respondents having a good sleep (34.8%).

#### Risk factors for academic-related fatigue

Bivariate correlations between the observed variables are presented in Table 1. The pattern of associations between the main study variables was generally in line with expected. Fatigue was significantly and positively related to a negative coping style, psychological detachment, negative emotion, workload, stress, physical health, and academic-life balance. Academic-related fatigue was significantly and negatively associated with lifestyles, positive well-being, and academic satisfaction.

**Table 1:** Bivariate correlations between observed variables.

Variables	1	2	3	4	5	6	7	8	9	10	11	12	13	14	15	16	17
1. Fatigue	1																
2. Positive personality	−0.063	1															
3. Positive coping style	0.002	0.553**	1														
4. Negative coping style	0.297**	−0.165**	−0.160**	1													
5. Self-efficacy	−0.057	0.708**	0.603**	−0.112**	1												
6. Psychological detachment	0.204**	0.250**	0.361**	0.021	0.298**	1											
7. Lifestyle	−0.141**	0.425**	0.369**	−0.080*	0.389**	0.201**	1										
8. Negative emotion	0.334**	−0.074*	0.03	0.370**	−0.089*	0.118**	−0.103**	1									
9. Workload	0.496**	0.037	0.058	0.249**	0.026	0.280**	−0.039	0.258**	1								
10. Stress	0.594**	−0.105**	−0.018	0.261**	−0.170**	0.209**	−0.092*	0.374**	0.479**	1							
11. Social support	0.06	−0.185**	−0.189**	0.099**	−0.212**	−0.162**	−0.162**	0.071	0.085*	0.111**	1						
12. Environments	0.362**	0.003	−0.04	0.222**	−0.003	0.116**	−0.095**	0.129**	0.256**	0.263**	0.048	1					
13. Physical health	0.627**	−0.092*	−0.011	0.308**	−0.108**	0.164**	−0.176**	0.299**	0.438**	0.397**	0.087*	0.401**	1				
14. Positive well-being	−0.100**	0.720**	0.601**	−0.164**	0.765**	0.280**	0.462**	−0.06	0.024	−0.139**	−0.185**	−0.042	−0.149**	1			
15. Academic-life balance	0.489**	−0.204**	−0.191**	0.462**	−0.249**	0.106**	−0.214**	0.415**	0.391**	0.463**	0.089*	0.324**	0.512**	−0.259**	1		
16. Academic efficacy	0.041	0.508**	0.566**	−0.087*	0.680**	0.388**	0.337**	−0.019	0.128**	−0.081*	−0.134**	0.017	0.012	0.588**	−0.175**	1	
17. Academic satisfaction	−0.092*	0.439**	0.473**	−0.029	0.611**	0.178**	0.237**	−0.032	−0.033	−0.214**	−0.134**	−0.016	−0.098**	0.566**	−0.201**	0.589**	1

Two-tailed testing of significance, *N* = 756, ^*^p < 0.05; ^**^p < 0.01.

The predictors of academic fatigue were then examined by carrying out a hierarchical multiple regression analysis. According to the variance inflation factor and tolerance statistics, there was no multi-collinearity among the independent variables in any regression models. Results are summarized in Table 2. In step 1 of the analysis, individual differences accounted for 11.5% of the variance in academic-related fatigue (*R^2^* = 0.115, *p* < 0.001), with the coping style, psychological detachment, emotion, and lifestyle as predictors (*p* < 0.001). The inclusion of academic characteristics in step 2 accounted for an additional 20.8% of the variance in academic fatigue (*R^2^* change = 0.208, *p* < 0.001), with the workload and stress (*p* < 0.001) as the significant predictors. In the last two steps of the analysis, the learning environment accounted for an additional 3.6% of the variance in academic fatigue (*R^2^* change = 0.036, *p* < 0.001), with the disturbed or noisy surroundings as a significant predictor (β = 0.199, *p* < 0.001). It should be noted that individual differences, including coping style and lifestyle, as well as academic characteristics, presented as the most common significant predictors of fatigue in each step of the analysis.

**Table 2:** Hierarchical multiple regression analysis for risk factors of academic-related fatigue.

	β			
	Step 1	Step 2	Step 3	Step 4
Individual difference				
Coping style	0.130^***^	0.081^*^	0.082^*^	0.074^*^
Self-efficacy	−0.06	−0.032	−0.034	−0.028
Psychological detachment	0.165^***^	0.045	0.043	0.043
Emotion	0.167^***^	0.043	0.042	0.044
Lifestyle	−0.151^***^	−0.131^*^	−0.131^***^	−0.094^**^
Academic characteristics				
Workload	–	0.232^***^	0.232^***^	0.198^***^
Stress	–	0.364^***^	0.365^***^	0.359^***^
Academic source				
Social support	–	–	−0.012	−0.015
Learning environment				
Noise and fumes	–	–	–	0.199^***^
*R^2^*	0.115	0.323	0.323	0.359
*F*	19.54^***^	51.00^***^	44.59^***^	46.52^***^

β, standardized coefficient; ^*^p < 0.05; ^**^p < 0.01; ^***^p < 0.001.

#### Adverse outcomes associated with fatigue

To understand the adverse consequences of various fatigue among college students, thresholds were used to dichotomize the variables into low and high fatigue groups, and an independent *t*-test was conducted. Results (see Table 3) showed that fatigue may have more negative effects on students’ physical health but no significant effect on well-being or academic performance. More specifically, students with higher academic fatigue reported poor physical health (*t* = −16.08, *p* < 0.001), and lack of academic-life balance (*t* = −11.04, *p* < 0.001) than those with low academic fatigue.

**Table 3:** Individual health outcomes under the different levels of academic fatigue.

	Low fatigue		High fatigue		*t*
	*M*	*SD*	*M*	*SD*	
Physical health	3.28	2.11	5.83	2.19	−16.08^***^
Well-being	6.52	1.76	6.35	1.66	1.32
Academic-life balance	3.78	2.36	5.70	2.324	−11.04^***^
Academic efficacy	5.58	1.66	5.78	1.80	−1.58
Academic satisfaction	5.25	2.21	4.98	2.30	1.61

***p < 0.001.

#### The relationships between fatigue and sleep quality

Table 4 presents the means, standard deviations, and bivariate correlation analyses of fatigue and various aspects of sleep scores. As expected, fatigue was significantly and positively related to self-reported sleep quality, sleep latency, sleep duration, habitual sleep efficiency, sleep disturbances, daytime dysfunction, and global PSQI scores. There was no significant association between fatigue and the use of sleeping medication.

**Table 4:** Means, standard deviations, and correlation coefficient matrix for each variable.

	1	2	3	4	5	6	7	8	9
1. Fatigue	1								
2. Subjective sleep quality	0.258^**^	1							
3. Sleep latency	0.245^**^	0.445^**^	1						
4. Sleep duration	0.178^**^	0.275^**^	0.161^**^	1					
5. Sleep efficiency	0.107^**^	0.171^**^	0.168^**^	0.522^**^	1				
6. Sleep disturbances	0.253^**^	0.476^**^	0.415^**^	0.196^**^	0.160^**^	1			
7. Use of sleeping medication	0.066	0.184^**^	0.129^**^	0.054	0.003	0.228^**^	1		
8. Daytime dysfunction	0.323^**^	0.470^**^	0.367^**^	0.243^**^	0.108^**^	0.456^**^	0.139^**^	1	
9. Overall PSQI score	0.354^**^	0.718^**^	0.680^**^	0.560^**^	0.498^**^	0.685^**^	0.292^**^	0.725^**^	1
*M*	4.98	1.93	0.87	0.41	0.35	0.88	1.06	1.42	6.93
*SD*	2.45	0.69	0.92	0.64	0.75	0.66	0.35	1.06	3.17

PSQI, Pittsburgh sleep quality index; **p < 0.01.

To assess associations between fatigue shaped and sleep quality among college students, an independent *t*-test was also conducted, and the results showed a significant difference between groups. Compared to those with lower fatigue, students with higher academic fatigue reported poor sleep quality (*t* = −5.60, *p* < 0.001) and sleep latency (*t* = −5.31, *p* < 0.001), shorter sleep duration (*t* = −4.05, *p* < 0.001), more sleep disturbances (*t* = −5.47, *p* < 0.001), and more severe daytime dysfunction (*t* = −7.22, *p* < 0.001). Higher fatigue indicated a high full PSQI score (*t* = −7.52, *p* < 0.001) as well, indicating a worse sleep quality they lead. However, neither sleep efficiency nor the use of sleeping medications was found to make a significant difference (*p* > 0.05).

#### Gender differences in sleep quality and fatigue among college students

To understand the difference in sleep quality between male and female college students, an independent samples *t*-test was conducted, and the results (Table 5) showed that there was a significant difference between the sleep quality of male and female college students, specifically, the sleep quality of female college students was significantly worse than that of male college students (*t* = −4.987, *p* < 0.001).

**Table 5:** *T*-test of sleep quality among male and female college students.

	Male		Female		
	*M*	*SD*	*M*	*SD*	*t*
Sleep quality	1.84	0.679	2.09	0.713	−4.987***

***p < 0.001.

Table 6 presents the differences in academic fatigue feelings between male and female college students, and the results show that there are significant differences between male and female college students’ fatigue feelings, with female college students’ fatigue feelings being significantly higher than those of male college students.

**Table 6:** *T*-test of academic fatigue among male and female college students.

	Male		Female		
	*M*	*SD*	*M*	*SD*	*t*
Academic fatigue	4.68	2.424	5.58	2.373	−6.628***

***p < 0.001.

### Summary

Study 1 was conducted to evaluate the prevalence, risk factors, and adverse outcomes associated with fatigue among Chinese college students. It established the existence of academic fatigue among college students, uncovered the possible influencing factors, and provided some preliminary evidence for the association between academic fatigue and poor sleep quality. Our study revealed that academic fatigue is common and that individual differences, including coping styles and lifestyle, as well as academic characteristics, were found to be significant factors associated with such fatigue. Given that fatigue in college students is closely related to poor physical health, lack of academic-life balance, and poor sleep quality, it is suggested that the aforementioned risk factors require close attention when developing a preventive intervention.

In line with previous studies (Tsai and Li, 2004; Calaguas, 2011; Bani-issa et al., 2018), there were gender differences in academic fatigue and sleep quality among college students in this study, with female college students experiencing higher levels of academic fatigue and poorer sleep quality than males. In the online questionnaire, 57.86% of female college students reported high academic stress compared to 37.81% of male college students; nearly a quarter of female college students reported “poor” (20.07%) or “very poor” (3.34%) sleep quality. The average sleep efficiency of female college students who participated in the exercise intervention was about 80% on the starting day, but according to Hirshkowitz et al. (2015) and Ohayon et al. (2017), healthy individuals should achieve more than 85% sleep efficiency per night. All these phenomena indicate that the academic fatigue status and sleep problems in the female college population cannot be ignored. This provides the basis for our follow-up intervention study.

## Study 2

We know from previous studies that females are usually higher than males in terms of fatigue and stress perception (Calaguas, 2011). Also, the quality of sleep is worse in female students compared to male students (Tsai and Li, 2004; Regestein et al., 2010). In addition, based on the results of Study 1, we also found differences in perceived academic fatigue and sleep quality between male and female college students in this study. Specifically, female college students had higher fatigue perceptions than males and poorer sleep quality than males. Thus, on the one hand, we believe that selecting female college students as subjects for the intervention is more effective in showing where the effects of short-term exercise interventions lie, and on the other hand, there have been few studies of exercise interventions for fatigue or sleep that specifically focus on females, and we believe that such a study would provide a more targeted perspective. In summary, we conducted a follow-up intervention study focusing on college women based on the results of Study 1.

In Study 2, participants were taking a short-term exercise intervention for 6 days, and they performed 30 min of physical activity every day with moderate intensity. This study measured the sleep-related indicators using actigraphy and measured the change in the student’s perception of academic fatigue before and after the exercise intervention by using an online diary. The main aim of this study was to investigate whether a short-term exercise intervention affected academic fatigue and sleep problems in a typical academic fatigue group of female college students.

### Methods

#### Participants

We used G*Power to calculate the sample size, effect size *f* = 0.25, α = 0.05, 1-β = 0.8, and calculated the required sample size to be 33. Based on the calculated subject size, 36 female participants were recruited for this study. All of them were (1) female students from the universities in Shenzhen, China, (2) with no previous exercise habits, (3) self-reported academic fatigue as high (7–10 points) in SWELL, and (4) self-reported sleep problems in the Pittsburgh Sleep Questionnaire. During the experiment period, participants were strictly requested to avoid the use of tobacco, alcohol, caffeine and sleep-aiding drugs (sleeping pills, melatonin) to reduce the influence of those factors on the sleep results. Twenty-nine participants completed the experiment, including 21 participants in the exercise intervention group and 8 in the control group.

#### Sleep measurement

The present study used both subjective and objective measurements of sleep. The subjective measurement used was a sleep diary, while the objective measurement used was Actigraphy (Actigraph wgt3x-bt, United States). The sleep diary, including pre-diary and post-diary, mainly recorded the time they went to bed, subjective feelings about sleep quality, and fatigue before and after sleep.

Actigraphy is a non-invasive tool for monitoring human rest/activity cycles, which can be worn on the wrist. It allows data to be collected over several days in a non-laboratory dynamic environment to examine how sleep changes over time in daily life. Previous studies showed that actigraphy is a valid and reliable method of sleep assessment (Ancoli-Israel, 2003) and has become an accepted tool in sleep research (Sadeh, 2008). The main sleep parameters actigraphy measured in the present study were the total sleep time, sleep efficiency, and length of sleep. The actigraphy algorithm used in this experiment was the Sadeh algorithm (Ancoli-Israel et al., 2003) for young and middle-aged people, as the participants in this study were young college students.

#### Procedure

After discussing the research objectives and informed consent, participants in the experimental and control groups gave self-reports of academic fatigue and related health indicators. Participants’ height, weight, handedness, race, and wearing position were entered into each individual’s actigraphy at the beginning of the experiment to ensure accurate and individualized sleep measurements.

A randomized controlled design was used for the experiment. For the exercise intervention group for 6 days (from Sunday of the previous week to Friday of the following week), they were required to perform 30 min of moderate intensity, any type, frequency of physical activity during the day. They were also asked to upload a screenshot of their exercise time and the exercise program recorded in the mobile app in the “Exercise check-in” group chat by 24:00 each day. The control group was told not to exercise during the day. All participants were asked to wear actigraphy each night before going to bed and to take it off when they woke up the next day. At the end of the experiment, all participants returned the actigraphy and again performed a self-report of academic fatigue and related health indicators. A final data check, data entry and actigraphy initialization were performed for that round.

#### Statistical analysis

This study mainly used IBM SPSS for Windows, version 25. to analyze the data of the Academic Fatigue Perception Questionnaire and Actigraphy data before and after the experiment. A repeated measures analysis of variance (ANOVA) was used to determine whether the sleep status of female college students improved with the increase of the intervention time during the exercise intervention cycle. Also, to find out whether there were significant differences in academic fatigue perception, academic concentration, and changes in fatigue-induced health indicators (dizziness and headache, back pain) before and after exercise in the exercise intervention group of female college students. Finally, the independent samples *t*-test was used to analyze whether there was a significant difference in the degree of academic fatigue perception between female college students in the exercise intervention group and the control group.

### Results

#### Actigraphy data analysis

As shown by the between-group effect, *F* = 34.166, *p* < 0.001 in Table 7, there was a significant difference between the exercise intervention group and the control group in the length of time spent asleep. The within-group effect, *F* = 12.444, *p* < 0.001, indicated that there was a significant difference in the length of sleep measured at different times. The interaction effect, *F* = 15.9, *p* < 0.001, shows that there was a significant interaction between exercise intervention and time on the length of time spent asleep.

**Table 7:** Repeated measures analysis of variance for the length of sleep.

		*MS*	*df*	*F*	*p*
Between-group effects	Group	49000.938	1	34.166	0.000***
Within-group effects	Time	4250.221	3.315	12.444	0.000***
Interaction effects	Group* Time	5430.571	3.315	15.9	0.000***

MS, mean square; ^***^p < 0.001.

**Figure 1:**
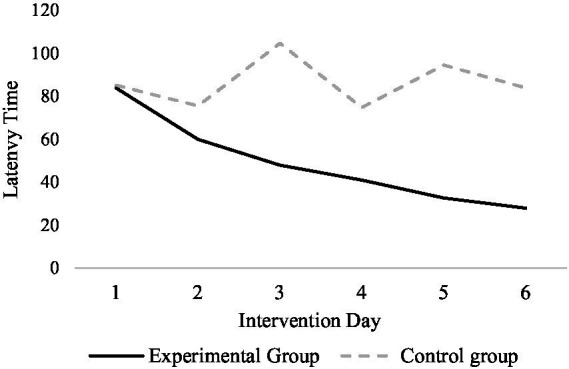
Comparison of the overall mean of the length of sleep in the experimental and control group.

The mean values of total sleep duration for the exercise intervention and control groups within each intervention day are shown in Figure 1. The overall mean value of the length of sleep in the exercise intervention group decreased gradually over the duration of the intervention, while there was no significant change in the control group. There was a significant difference in the length of time to fall asleep between female university students who had the exercise intervention and those who did not have any intervention, with students who had the exercise intervention falling asleep for a shorter period compared to those who did not have any intervention, and the length of time to fall asleep for female university students tended to decrease as the duration of the intervention increased in the exercise intervention condition.

As shown in Table 8, on the between-group effect, *F* = 6.121, *p* < 0.05, there was a significant difference between the exercise intervention group and the control group for total sleep duration. For the within-group effect, *F* = 4.651, *p* < 0.05, there was a significant difference in total sleep duration measured at different times. On the interaction effect, *F* = 1.244, *p* > 0.05, it can be seen that there was no significant interaction effect between exercise intervention and time for total sleep duration.

**Table 8:** Repeated measures analysis of variance for total sleep time.

		*MS*	*df*	*F*	*p*
Between-group effects	Group	25475.148	1	6.121	0.02*
Within-group effects	Time	11210.765	3.652	4.651	0.002**
Interaction effects	Group* Time	2997.358	3.652	1.244	0.298

MS, mean square; ^*^p < 0.05, ^**^p < 0.01.

**Figure 2:**
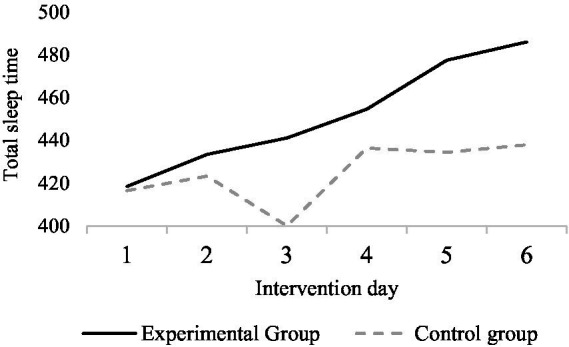
Comparison of the overall mean of total sleep time in the experimental and control groups.

The mean values of total sleep duration for the exercise intervention group and the control group within each intervention day are shown in Figure 2. The overall mean value of total sleep duration for the exercise intervention group gradually increased over time, while the control group showed fluctuations, with a small overall mean increase compared to the first day. There was a significant difference in total sleep duration between female university students who underwent the exercise intervention and those who did not undergo any intervention, with longer total sleep duration for the exercise intervention compared to those who did not undergo any intervention and an increasing trend in total sleep duration for those who underwent the exercise intervention as the duration of the intervention increased.

As shown in Table 9, for the between-group effect, *F* = 24.796, *p* < 0.001, it can be seen that there was a significant difference between the exercise intervention group and the control group for sleep efficiency. The within-group effect, *F* = 3.218, *p* < 0.05, shows a significant difference in sleep efficiency measured at different times. The interaction effect, *F* = 11.971, *p* < 0.001, between the exercise intervention and time, was significant for sleep efficiency.

**Table 9:** Repeated measures analysis of variance for sleep efficiency.

		*MS*	*df*	*F*	*p*
Between-group effects	Group	1887.798	1	24.796	0.000***
Within-group effects	Time	96.838	3.417	3.218	0.021*
Interaction effects	Group* Time	360.193	3.417	11.971	0.000***

MS, mean square; ^*^p < 0.05, ^***^p < 0.001.

**Figure 3:**
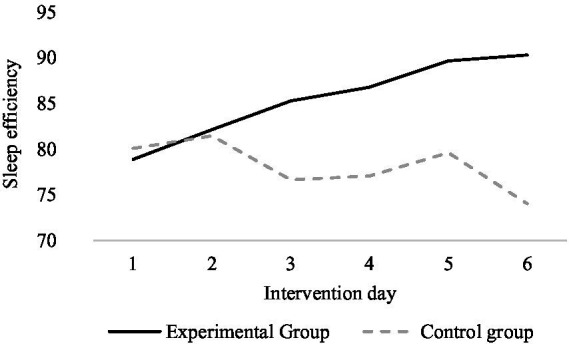
Comparison of the overall mean of the sleep efficiency of the experimental and control groups.

The mean values for sleep efficiency for the exercise intervention group and the control group within each intervention day are shown in Figure 3. The overall mean value of sleep efficiency for the exercise intervention group gradually increased with the duration of the intervention, while the control group showed fluctuations and a certain decrease compared to the overall mean value on the first day. There was a significant difference in sleep efficiency between the female university students who had the exercise intervention and those who did not have any intervention, with higher sleep efficiency in the exercise intervention compared to those who did not have any intervention and an increasing trend of greater improvement in sleep efficiency in the exercise intervention condition as the duration of the intervention increased.

#### Academic fatigue before and after the intervention

As shown in Table 10, the repeated measures ANOVA showed that there was a significant difference in the perception of academic fatigue before and after the experiment, *F* = 40.888, *p* < 0.001. There was also a significant difference in the dizziness and headache variable, *F* = 38.979, *p* < 0.001, and the back pain rating (*F* = 26.385, *p* < 0.001). In contrast, there was no significant difference in academic concentration before and after the experiment.

**Table 10:** Repeated measures ANOVA of fatigue perception, concentration, dizziness and headache, and back pain of participants in the intervention group before and after the experiment.

		*MS*	*F*	*p*
Within-group effects	Feelings of fatigue	127.841	40.888	0.000***
	Concentration	0.023	0.004	0.948
	Dizziness and headache	192.364	38.979	0.000***
	Back pain	111.364	26.385	0.000***

MS, mean square; ^***^p < 0.001.

The results (see Table 11) indicated that for the female college subjects who underwent the exercise intervention, the pre-experimental fatigue was significantly higher than the post-experimental fatigue. In addition, the positive effect of the exercise intervention may be accompanied by a reduction in the feelings of academic-induced dizziness and headache and back pain. However, there was no significant change in the improvement of academic concentration before and after the exercise intervention.

**Table 11:** Comparison of fatigue feeling, concentration, dizziness and headache, and back pain in the experimental group before and after the intervention.

		*M*	*SD*
Feelings of fatigue	Before	8.48	1.184
	After	5.45	2.197
Concentration	Before	5.79	1.859
	After	5.86	1.846
Dizziness and headache	Before	5.62	2.871
	After	4.34	2.511
Back pain	Before	7.59	2.413
	After	6.03	2.528

The results showed that there was no significant difference in the degree of academic fatigue felt by the female college subjects who underwent exercise intervention and those who did not undergo any intervention at the end of the experiment (see Table 12).

**Table 12:** Independent samples *t*-test between experimental and control groups on fatigue perception.

		*F*	Sig.	*t*	*df*	*p*
Fatigue perception	Assuming equal variance	0.027	0.87	−0.757	27	0.456
	Not assuming the isotropic variance			−0.77	10.413	0.459

### Summary

Study 2 specifically examined the effects of an exercise intervention on academic-related fatigue and sleep problems in female university students and analyzed the effects of a short-term exercise intervention using sleep measures and measures of perceived academic fatigue. The results showed that after 6 days of moderate intensity, any form of daily cumulative 30 min of exercise, these female college students with high levels of fatigue and sleep problems showed more significant improvements in the two indicators of sleep latency and sleep efficiency. In terms of fatigue perception, subjective reports indicated that the participants’ fatigue perception was reduced to some extent. Overall, the short-term exercise intervention resulted in a significant improvement in sleep quality and a decrease in perceived academic fatigue in female college participants.

## Discussion

In light of intense competition for employment, Study 1 investigated if fatigue is common and severe in emerging adulthood and, more specifically, in a sample of college students. The college stage is a critical period in life for development, and the healthy growth of young people meant much to individuals, families, and society. An enhanced understanding of the prevalence and predictors of academic fatigue among college students will help guide intervention development.

Consistent with previous research (e.g., Schaufeli et al., 2002), the current study found a moderate level of fatigue in college students, and two in five of this sample reported high fatigue. The results showed that applications for studying abroad, postgraduate entrance exams, and graduation thesis were the most important causes of academic fatigue. In general, all these situations are time-limited and require a great deal of energy and time, thereby creating great challenges for college students. Those who aspire to high achievements must exert a lot of effort, depleting themselves in a short time and causing inevitable fatigue. Moreover, most students surveyed rated themselves as having negative personalities, and about half thought they had an unhealthy lifestyle. The links between an unhealthy lifestyle and fatigue have been reported extensively in the literature (e.g., Bültmann et al., 2002; Lee et al., 2007), and this result paves the way for future intervention research.

Second, we analyzed the risk factors associated with academic fatigue among college students. In line with previous workplace research (Mark and Smith, 2011, 2012; Fan and Smith, 2020), the results suggest that most of these factors were positively associated with fatigue. We used hierarchical multiple regressions to confirm the factors that shape academic fatigue. Our results suggest that individual differences, academic characteristics, and learning environment interact to affect fatigue, and academic characteristics account for most of the effect, while the learning environment accounts for the least. In this way, we suggested that modifiable factors (e.g., lifestyle) should be emphasized when developing a preventive intervention. For example, encourage students to exercise more to reduce sedentary time, thereby reducing academic fatigue. It should be noted that the association between social support and fatigue in this study was significant, but it did not play a decisive role. Young adults in college may be relatively independent, and the lack of gain of social support has a relatively small impact.

Finally, the adverse consequences associated with fatigue were evaluated, and consistent with previous studies, it was found that higher academic fatigue leads to poor physical health and a lack of academic-life balance. Surprisingly, there was no significant effect on well-being, academic efficacy, or academic satisfaction in our study. A possible explanation is that Chinese students regard their academic events as their affairs and feel that the fatigue brought upon them will not affect their academic satisfaction. Presumably, the lack of academic-life balance, meaning that too much time was spent on academic events, does not affect academic efficiency. Of the many health outcomes that are associated with fatigue (Dawson et al., 2021), sleep quality deserves particular attention as sleep issues may impact individuals with externalizing psychopathology, as well as increase the potential for antisocial behavior (Taub and John, 1977; Kamphuis et al., 2012; Buysse, 2014). The assessment of the PSQI allowed us to examine whether fatigue would have an impact on sleep. In general, apart from the sleep efficiency and sleeping medications utilized, fatigue was associated with both the subscale and the overall score of PSQI. Even though there is now a longstanding body of research highlighting that insufficient sleep causes fatigue (Angus and Heslegrave, 1985), the current study, in line with many studies focused on fatigue-related impairment (Åkerstedt et al., 2002; Rodrigue et al., 2010), revealed that fatigue is in turn associated with poor sleep quality. This finding partially supported the perspective that poor sleep quality is closely related to both subjective cognitive decline and subjective cognitive decline-related functional limitations (Bradley et al., 2019).

Female college students were selected from Study 1 to participate in an exercise intervention to reduce their academic fatigue and improve their sleep quality. The results demonstrated that a short-term, arbitrary intensity and form of exercise intervention was effective in reducing academic fatigue and improving sleep quality among college students. This is a very efficient way for students with busy academic schedules. Previous studies have shown that balance training and yoga training have been shown to have a moderate alleviating effect on low back pain in people (Nambi et al., 2014; Brämberg et al., 2017; Susan and Wieland, 2017). In the present study, female university students often chose to use forms of exercise such as Pamela’s dance exercises and elliptical machines, which are effective in stretching the whole body and improving limb balance, and were, therefore, more likely to report a reduction in low back pain at the end of the experiment. In addition, exercise was shown to have a positive effect on sleep, with significant improvements in length of sleep and sleep efficiency. In line with established research (Li and Liu, 2011), improving sleep quality was associated with an increase in average daily activity, and this study suggests that a short-term exercise intervention was effective in reducing the length of sleep and improving sleep efficiency in female university students. At the end of the experiment, the difference in mean sleep efficiency between the experimental and control groups was around 15%, and the difference in sleep duration was around 40 min, demonstrating the effectiveness of the short-term exercise intervention.

But some of the results obtained in this study differ from established research, and this needs further discussion. Firstly, the reduction in academic fatigue in the exercise intervention group was not necessarily solely caused by exercise, and other influences may have been present. The study has shown that the perception of stress-induced fatigue in the daily lives of university students fluctuates over time as life events unfold (Chen-Ling, 2002), and therefore academic fatigue may also change over time. Due to the lengthy and extensive process of the experiment, a total of 3 months elapsed between the online questionnaire and the conduct of the exercise intervention experiment. It is possible that during these 3 months, there was a change in the participant’s academic fatigue due to changes in academic tasks, and this unavoidable phenomenon may have influenced the results.

Similarly, the increase in total sleep time may have been caused by other factors. For example, due to the phenomenon of ‘social jet lag’, common among students (Castilhos Beauvalet et al., 2017), where sleep patterns between weekdays and weekends do not coincide, subjects sleep longer on Friday nights; research suggests that school hours limit adolescents’ weekday sleep (Elfering et al., 2020) and that subjects may have early classes on weekdays. The participants may have early morning classes scheduled on weekdays or may not have classes throughout the morning; therefore, this situation may have a greater impact on the subjects’ total sleep duration.

The present study also focused on aspects that have been less addressed in previous studies, providing a novel perspective on how exercise interventions are delivered and how sleep is measured. It is worth noting that most previous studies have focused on exercise interventions of more than 6 months compared to short-term exercise interventions of 6 days (Fernandes et al., 2006; Li and Liu, 2011), and this study demonstrates the feasibility of short-term exercise interventions as a quick and effective way to improve sleep quality and reduce feelings of fatigue in female university students. In this experiment, the exercise intervention was based on “30 min of moderate intensity, any type of physical activity per day,” and the volunteers mostly used the school gym, school field, playground running, and dormitory gymnastics as exercise punch cards. These forms of exercise are not restricted by location and can make full use of free and fragmented time. Therefore, this type of exercise intervention is more operational for university students with a large academic load and is more likely to help them develop exercise habits that will lead to lasting improvements in their physical and mental health. In addition, actigraphy was used in this study to continuously monitor the sleep of female university students over 6 days, which provided an important safeguard for obtaining accurate sleep data for this study. As an ambulatory, non-invasive sleep measurement instrument (Sadeh, 2011), the actigraphy helps to increase the ecological validity of the results, and the experiment with the actigraphy in this study provides a reference for research ideas for its further dissemination in China.

## Limitations and future study

Previous fatigue studies have been mainly conducted in workplaces. This study assessed the fatigue status among university students and identified factors that influenced it from a more comprehensive perspective. In addition, the effects of daily academic fatigue on youth’s health were analyzed, especially those associated with sleep quality. The findings extend the understanding of fatigue, its risk factors, and its associations with sleep and health problems in Chinese young adults. It also provides a simple and efficient way for Chinese university students to relieve fatigue and improve sleep quality.

However, the studies had several limitations. First, all measures used in Study 1 were self-reported questionnaires, which may lead to an overestimation or underestimation of the sense of fatigue. However, before the study, all the participants were told to answer the questionnaires as accurately as possible and to avoid thinking too much about their answers. Secondly, participants in this study included only students living in the south of China, and the education and academic ability levels may be different in other regions. Therefore, the generalization of our conclusions to all university students should be made with caution. How these results might vary across countries could be determined in further study. Finally, Study 1 was a cross-sectional design performed over a month, which makes it hard to establish causality. Additional studies are necessary to further explore these questions of causality by using a longitudinal design.

Meanwhile, although Study 2 provides a new perspective on the approach to exercise intervention and sleep measurement and focuses on a typical group of female college students, there are some shortcomings in this study as well. First, one of the more obvious problems is the small sample size. Due to the limited experimental period and the limited number of eligible subjects screened by the online questionnaire, there were 36 female college subjects in the exercise intervention experiment, and after removing invalid data from 7 subjects, a total of 29 subjects’ data were involved in the data analysis, and the smaller sample size limited the effect size of certain statistics (Heidel, 2016).

In addition, as a social experiment, Study 2 used several methods in supervising volunteers, but it was still difficult to ensure complete control of irrelevant variables. First, before the start of the intervention experiment, the experimenter demonstrated how to wear the actiwatch and created detailed instructions for the experiment, and created an “exercise check-in group” for each experimental period, where daily exercise punch cards were monitored, and reminders were given to wear the actiwatch before sleep. Despite this, seven participants failed the data collection due to wearing errors or forgetting to wear the actiwatch on one night. Secondly, although the experimental instructions clearly stated that the use of alcohol, tobacco, stimulant drinks, and sleep-aid drugs was strictly prohibited, it was difficult to guarantee that participants would not drink or smoke in private. Thirdly, there are additional factors affecting the sleep duration of college students that are not found in typical occupational fatigue groups. The phenomenon of “social jet lag” mentioned in previous studies (Castilhos Beauvalet et al., 2017) suggests that the frequent use of electronic devices and the abundance of social activities contribute to the unstable sleep patterns of students. In the present experiment, individuals also reported excessive night-time cell phone use up to 3:00 a.m., club activities and dinners leading to an early morning return to the dormitory. Fourthly, the schedule of weekday morning classes also had an impact on their total sleep time; for example, those who needed to attend one or two-morning classes had a restricted total sleep time. In summary, although the study had many factors that are difficult to control, the interaction of these factors with the study variables also increases the ecological validity of the experiment, and this is one of the characteristics of social experiments.

Finally, there are some limitations of sleep measurement through actigraphy. On the one hand, although actigraphy is widely used in sleep measurement as an accurate sleep instrument (Sadeh, 2008), it still has some limitations. First, actigraphy is an indirect measurement that estimates sleep–wake time mainly by measuring the activity of the body and, therefore, may have some errors. Second, actigraphy cannot capture some important sleep parameters, such as the percentage of time spent in light sleep, deep sleep, and REM sleep, which are good indicators of sleep to distinguish the experimental and intervention groups (Kobayashi et al., 2007). In addition, the sleep measurement cycle conducted in this experiment was 6 days, and the short measurement time makes it difficult to identify the complete and stable sleep cycle of the volunteers, thus making it difficult to fully grasp the changes in the individual’s stable sleep profile, as well as to understand the bi-directional relationship between fatigue and sleep.

Future research can be further improved based on this experiment. First, one could consider doubling the sample size, which will not only make the results more reliable but also make the validity of the study higher. If the sample size was larger, one could also consider classifying the exercise intervention group more carefully, such as whether they need to attend morning classes or not and whether they often participate in club activities or study groups during the experimental cycle or not. In addition, the practical operation of the actigraphy in Study 2 also requires attention to address possible problems such as wearing it incorrectly or forgetting to wear it, and future studies could write more specific and focused experimental instructions and adopt stricter monitoring measures to improve the data collection rate. In addition, previous studies have found that sleep is closely related to the respiratory activity and electrocardiographic activity (Ichikawa et al., 2008; Long et al., 2014). Therefore, it is possible to consider supplementing the actigraphy measurements with respiratory and electrocardiographic measurements to improve the accuracy of the data. In addition, to capture a more stable sleep cycle and to improve the overall understanding of the changes in individual sleep conditions, future studies may consider appropriately extending the duration of short-term exercise interventions. Finally, for subjective measures of academic fatigue, there may be a tendency for participants to give a biased view of themselves. Therefore, a combination of subjective and objective methods should be used in the future to reduce errors.

## Conclusion

This study was divided into two parts to explore academic-related fatigue and sleep problems among female college students. Study 1 explored gender differences in academic fatigue perceptions and sleep problems among male and female college students and concluded that female college students had higher academic fatigue perceptions than male college students and that female college students had higher rates of poor sleep caused by academic stress than male college students. Study 2 explored the effects of an exercise intervention on the perception of academic fatigue and sleep problems in female college students and showed that a short-term exercise intervention could help improve academic fatigue and sleep problems in female college students. This study focused on a group of female college students who have received less attention in fatigue studies so far, used actigraphy, which is not yet popular in China, to measure sleep-related indicators, and verified the feasibility of a short-term exercise intervention. Therefore, this study provides a feasible and convenient way for female college students to reduce academic fatigue and improve sleep.

## Data availability statement

The raw data supporting the conclusions of this article will be made available by the authors, without undue reservation.

## Ethics statement

The studies involving human participants were reviewed and approved by Shenzhen University. The patients/participants provided their written informed consent to participate in this study.

## Author contributions

JF formulated the research question and designed the study. JC conducted the data collection. WL and ML performed the data analysis and interpretation. WL drafted the manuscript. AS and JF provided critical revisions. All authors approved the final version of the manuscript for submission.

## Conflict of interest

The authors declare that the research was conducted in the absence of any commercial or financial relationships that could be construed as a potential conflict of interest.

## Publisher’s note

All claims expressed in this article are solely those of the authors and do not necessarily represent those of their affiliated organizations, or those of the publisher, the editors and the reviewers. Any product that may be evaluated in this article, or claim that may be made by its manufacturer, is not guaranteed or endorsed by the publisher.
